# GenoGlyph: Pan-cancer genomic mutation inference and risk stratification from diagnostic histopathology slides

**DOI:** 10.21203/rs.3.rs-10158096/v1

**Published:** 2026-07-09

**Authors:** Abdul Rehman Akbar, Usama Sajjad, Alejandro Leyva, Elshad Hassanov, Arya Mariam Roy, Daniel Stover, Wencheng Li, Ashish Manne, Wei Chen, Anil Parwani, Muhammad Khalid Khan Niazi

**Affiliations:** 1 Department of Pathology, College of Medicine, The Ohio State University Wexner Medical Center, Columbus, OH, USA; 2 Department of Internal Medicine, Division of Medical Oncology, The Ohio State University Wexner Medical Center, Columbus, OH, USA; 3 Department of Pathology, Wake Forest University School of Medicine, Winston-Salem, NC, USA

## Abstract

Genomic alterations drive therapeutic decisions in solid tumors, yet next-generation sequencing remains inaccessible in a substantial fraction of clinical settings worldwide. We present GenoGlyph, an interpretable deep learning framework designed to decode the grammar of histopathology for pan-cancer genomic mutational inference and clinical risk stratification. Across 6,391 whole-slide images spanning 14 solid tumor types, GenoGlyph robustly predicted actionable alterations (e.g., *TP53, KRAS, PIK3CA, APC*), yielding AUCs of 0.63–0.98 and outperforming existing pan-cancer benchmarks. To determine whether these morphologically inferred genomic signatures capture underlying tumor biology, we integrated mutation predictions with matched transcriptomic profiles and observed concordant activation of canonical pathways, such as elevated mTORC1 signaling in PIK3CA-mutated tumors. Furthermore, we demonstrate GenoGlyph’s utility as a functional genomics tool by showing that mutations predicted by the model, including sequencing-discordant (false-positive) cases, exhibit pathway perturbations consistent with the inferred genotype, such as suppression of DNA repair programs in TP53-mutated tumors. Finally, we validate the clinical utility of GenoGlyph’s learned representations by demonstrating independent prognostic value for overall, disease-free, recurrence-free, and progression-free survival across four independent external cohorts (n=982). GenoGlyph establishes that complex genotype-phenotype relationships are systematically legible from standard H&E sections. Rather than treating mutation prediction as an endpoint, our framework leverages these genomic signatures to learn latent features encapsulating hidden tumor-intrinsic and microenvironmental states. This provides a scalable, biologically grounded paradigm for democratized precision oncology, functional variant interpretation, and survival stratification.

## Introduction

Cancer care is undergoing a profound transformation driven by the integration of histopathology, genomics, and clinical decision-making. Across solid tumors, therapeutic selection, prognostic stratification, and eligibility for targeted therapies are increasingly dictated by specific genomic alterations, including mutations in canonical driver genes such as *KRAS, TP53, PIK3CA, BRAF*, and *APC* [1–7]. Consequently, comprehensive molecular profiling has become a cornerstone of modern oncology practice. However, access to next-generation sequencing (NGS) remains uneven across healthcare systems, limited by cost, infrastructure, tissue availability, and turnaround time, thereby creating disparities in timely and equitable delivery of precision oncology [8, 9]. In parallel, hematoxylin and eosin (H&E)–stained histopathology slides remain universally available, rapid to acquire, and deeply embedded in diagnostic workflows [10].

A growing body of evidence suggests that tumor morphology is not merely descriptive but reflects the downstream consequences of genomic and epigenomic alterations [11–13]. Oncogenic mutations drive changes in cellular proliferation, differentiation, stromal remodeling, immune infiltration, and metabolic states, all of which manifest as reproducible, although often subtle, histomorphological patterns [14–16]. Recent advances in artificial intelligence have leveraged this premise, demonstrating that deep learning (DL) models trained on digitized whole-slide images (WSIs) can predict selected genomic alterations directly from histology across multiple tumor types [12, 13, 17, 18]. These studies have established proof-of-concept that genotype–phenotype relationships are, at least in part, visually encoded. However, existing approaches largely treat histopathology as a collection of independent image patches, relying on weakly supervised learning paradigms that do not explicitly model the structured, hierarchical organization of tissues [19–34]. As a result, the biological interpretability, generalizability, and clinical utility of these models remain limited.

Histopathology is essentially a structured language where individual cells represent fundamental units, similar to words, with their identities determined by morphology and staining properties. [35, 36].. Their spatial arrangements and interactions form higher-order constructs like cellular neighborhoods, tissue architecture, stromal patterns, and immune niches that resemble phrases, sentences, and paragraphs. This hierarchical organization encodes complex biological information arising from tumor evolution and microenvironmental interactions. We posit that decoding this “language of histopathology” requires models that move beyond texture recognition to explicitly capture cellular composition, spatial context, and multi-scale organization.

Here, we present GenoGlyph, a DL framework designed to decode the language of histopathology for pan-cancer genomic inference and clinical risk stratification. Leveraging digitized H&E WSIs spanning diverse malignancies, GenoGlyph learns representations that integrate cellular morphology with spatial context across scales. The model is trained to infer mutations in clinically relevant genes, including *KRAS, TP53, PIK3CA, PBRM1, TTN, APC, PTGS2* (*COX2*), *CDKN2A, FAT1, CTNNB1, KMT2D, BRAF, NRAS*, and *ARID1A*, capturing diverse oncogenic pathways across tumor types. Beyond prediction, we validate biological concordance between GenoGlyph’s mutation predictions and matched transcriptomic pathway activity, including functional annotation of sequencing-discordant cases as candidate variants of uncertain significance (VUS).

**Figure 1 |:**
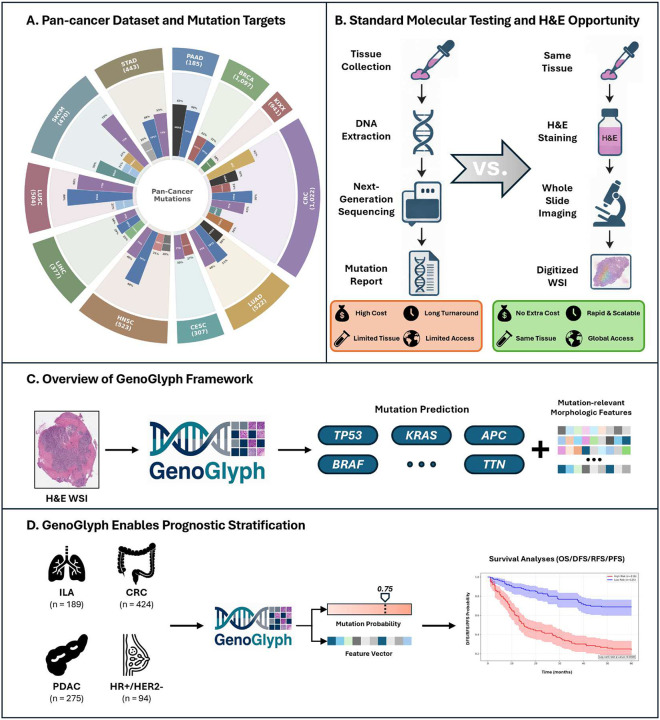
Overview of the GenoGlyph framework, study design, and pan-cancer cohort composition. Schematic overview of the GenoGlyph framework for pan-cancer mutation prediction and prognostic stratification from routine hematoxylin and eosin (H&E)-stained WSIs. The study included 6,391 WSIs spanning 14 solid tumor types from The Cancer Genome Atlas (TCGA). Cancer type-specific models were trained for 34 mutation prediction tasks across 14 genes, while organ-agnostic pan-cancer models were developed for *TP53, KRAS, APC, PIK3CA, KMT2D*, and *TTN*. Learned genomic-aware latent representations were subsequently evaluated for prognostic stratification across four independent external cohorts encompassing lung adenocarcinoma, colorectal adenocarcinoma, pancreatic ductal adenocarcinoma, and metastatic hormone receptor-positive/HER2-negative breast cancer.

Crucially, GenoGlyph reframes mutation prediction not as an endpoint but as a biologically grounded supervision signal for representation learning. By learning mutation-associated morphological patterns across cancers, the model constructs a latent space of “genomic-aware” histopathological features. These features encapsulate tumor-intrinsic properties and microenvironmental states that are otherwise inaccessible through conventional histomorphologic visual assessment. We subsequently demonstrate that these learned representations possess significant prognostic value, enabling prediction of overall (OS), disease-free (DFS), recurrence-free (RFS), and progression-free survival (PFS) across tumor types. In doing so, GenoGlyph establishes a direct link between histomorphology, genomic alterations, and patient outcomes within a unified framework (see Figure 1).

## Results

### Dataset composition and corpus characteristics

We used digitized H&E WSIs from The Cancer Genome Atlas (TCGA), spanning 14 solid tumor types including, pancreatic adenocarcinoma (PAAD), breast invasive carcinoma (BRCA), kidney clear cell carcinoma (KIRC), kidney renal papillary cell carcinoma (KIRP), kidney chromophobe (KICH), colon adenocarcinoma (COAD), rectal adenocarcinoma (READ), lung adenocarcinoma (LUAD), cervical squamous cell carcinoma (CESC), head and neck squamous cell carcinoma (HNSC), liver hepatocellular carcinoma (LIHC), lung squamous cell carcinoma (LUSC), skin cutaneous melanoma (SKCM), and stomach adenocarcinoma (STAD), totaling 6,391 cases [37].

For each cancer type, we selected mutation targets with a minimum prevalence threshold of 20% of mutated cases in that cohort, ensuring sufficient class representation for model training. This yielded 34 cancer type-specific mutation prediction tasks across 14 genes. Mutation and wild-type labels were derived from the data released by Kather et al., ensuring consistency with prior published benchmarks [13]. The resulting dataset composition and per-gene mutation frequencies are summarized in Figure 1.

In addition to cancer type-specific models, we trained pan-cancer, organ-agnostic models for six genes with sufficient representation across cohorts: *TP53* (4,107 cases), *PIK3CA* (3,091 cases), *TTN* (4,788 cases), *KRAS* (1,255 cases), *KMT2D* (2,199 cases), and *APC* (1,142 cases). These pan-cancer models were designed to test whether mutation-associated morphological signals generalize across tissue types and histological contexts, representing a more stringent evaluation of the genotype-phenotype relationships encoded in H&E morphology. We further evaluated these models under a leave-one-cancer-out (LOCO) protocol, where each cancer type was held out in turn during training and used exclusively for testing, providing a rigorous assessment of cross-tissue generalizability.

Furthermore, we assessed the six pan-cancer models as feature extractors for patient prognostic stratification across four independent multi-institutional cohorts (n = 982) encompassing distinct clinical contexts including, invasive lung adenocarcinoma (n = 189), colorectal adenocarcinoma (n = 424), pancreatic ductal adenocarcinoma (n = 275), and metastatic hormone receptor-positive, HER2-negative breast cancer (n = 94). Survival endpoints included OS, DFS, RFS, and PFS, with endpoint availability varying by cohort and clinical setting.

### GenoGlyph achieves robust mutation prediction performance across diverse tumor types

**Figure 2 |:**
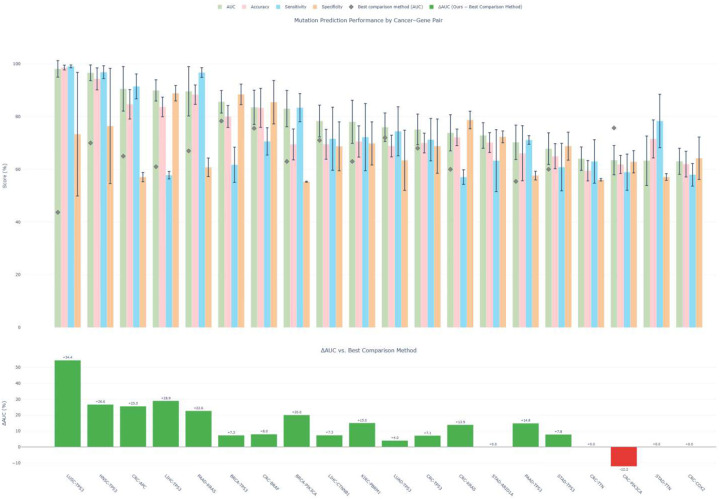
GenoGlyph mutation prediction performance on multiple gene prediction tasks across diverse tumor types. Grouped bar charts show the mean AUC, accuracy, sensitivity, and specificity (± standard deviation across cross-validation folds) for predicting somatic mutations in 20 cancer–gene pairs spanning several tumor types. Diamond markers (gray) indicate the best published AUC from prior methods for each cancer–gene pair. The lower panel shows the absolute difference in AUC between our model and the best comparison method (ΔAUC); green and red bars indicate improvement and underperformance, respectively.

**Figure 3 |:**
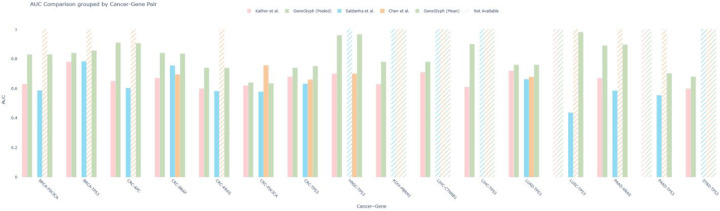
GenoGlyph mutation prediction performance comparison with previous benchmarks. Grouped bar charts show the mean or pooled AUC of previous benchmarks (reported as is from literature) compared with GenoGlyph’s mean or pooled AUC across several cancer-gene prediction tasks.

GenoGlyph accurately predicts diverse genomic alterations across multiple tumor types, demonstrating that mutation-associated histomorphological signals are broadly encoded within routine H&E WSIs, with predictive performance exceeding previously reported gene mutation prediction benchmarks [13, 38, 39] (Figures 2, 3; **Tables S9, S10**). Several gene-cancer pairs exhibited particularly strong discrimination, with TP53 mutations showing some of the highest predictive performance across multiple malignancies. TP53 prediction achieved AUCs of 0.98 ± 0.03 in LUSC and 0.97 ± 0.03 in HNSC, accompanied by accuracies of 98.63% and 94.32%, respectively. Strong performance was also observed for TP53 in LIHC (AUC 0.90 ± 0.04), BRCA (AUC 0.86 ± 0.04), CRC (AUC 0.75 ± 0.06), and LUAD (AUC 0.76 ± 0.05), highlighting the widespread morphological manifestations associated with this key tumor suppressor alteration.

Beyond TP53, several additional driver mutations demonstrated substantial predictability from H&E morphology. APC and BRAF mutations in CRC achieved an AUC of 0.91 ± 0.08 and 0.84 ± 0.07 respectively, while KRAS mutations reached an AUC of 0.90 ± 0.09 in PAAD, with particularly high sensitivity (96.72%), indicating effective identification of mutation-positive cases. PIK3CA mutations in BRCA also exhibited strong discrimination (AUC 0.83 ± 0.07). Strong predictive performance was additionally observed for PBRM1 mutations in kidney cancers (AUC 0.78 ± 0.08) and CTNNB1 mutations in LIHC (AUC 0.78 ± 0.06), supporting the presence of recognizable morphological phenotypes linked to these genomic alterations.

Moderate yet reproducible performance was observed for several other clinically relevant mutations, including KRAS in CRC (AUC 0.74 ± 0.07), ARID1A in STAD (AUC 0.73 ± 0.05), STAD TP53 (AUC 0.68 ± 0.06), PAAD TP53 (AUC 0.70 ± 0.07), and CRC COX2 (AUC 0.63 ± 0.05). Additional alterations, including PIK3CA and TTN in CRC and TTN in STAD, yielded AUCs ranging from 0.63 to 0.64. Collectively, these findings demonstrate that GenoGlyph can accurately identify mutation-associated morphological signatures across a broad spectrum of clinically relevant cancer–gene combinations, supporting the existence of pervasive genotype–phenotype relationships in solid tumors.

### Pan-cancer organ-agnostic models capture tissue-invariant mutation signatures and frequently surpass cancer type-specific counterparts

**Figure 4 |:**
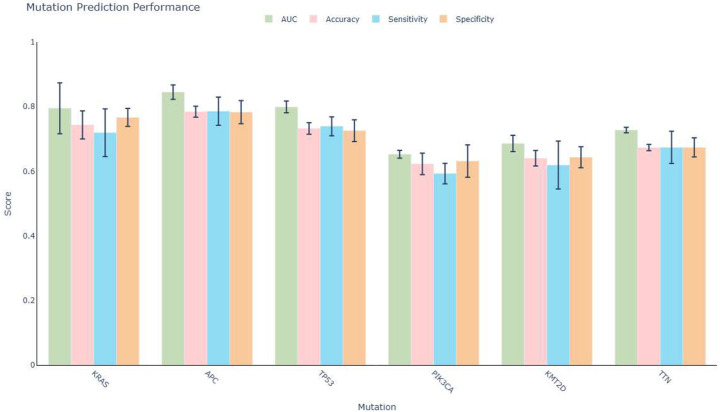
Pan-cancer organ-agnostic mutation prediction reveals transferable histomorphological signatures. Performance of organ-agnostic pan-cancer GenoGlyph models trained across multiple tumor types for six recurrently represented genes: *TP53, KRAS, APC, PIK3CA, KMT2D*, and *TTN*. Models were trained by pooling cases from all eligible cancer types for each gene, thereby requiring the network to distinguish mutation-associated morphology independently of tissue-specific histological context. Bar plots summarize mean AUC values across five-fold patient-level cross-validation, with error bars representing standard deviation.

To evaluate whether GenoGlyph can learn mutation-associated morphological patterns that generalize across histological contexts, we trained six organ-agnostic pan-cancer models, one per gene, pooling cases across all cancer types in which each mutation was represented: *TP53* (n = 4,107; 10 cancer types), *TTN* (n = 4,788; 14 cancer types), *PIK3CA* (n = 3,091; 7 cancer types), *KRAS* (n = 1,255; 4 cancer types), *KMT2D* (n = 2,199; 7 cancer types), and *APC* (n = 1,142; 4 cancer types). This represents a substantially more stringent evaluation regime than cancer type-specific training, as the model must disentangle mutation-associated morphology from organ-specific histological variation (Figure 4, **Table S6**).

At the pan-cancer level, *APC* achieved the strongest overall discrimination (AUC 0.85 ± 0.02), followed by *TP53* (AUC 0.80 ± 0.02) and *KRAS* (AUC 0.80 ± 0.08). *TTN*, despite being a passenger mutation, reached an AUC of 0.73 ± 0.01, while *KMT2D* and *PIK3CA* demonstrated modest pan-cancer performance (AUC 0.69 ± 0.02 and 0.65 ± 0.01, respectively). Notably, the reduced variance across folds for pan-cancer models, relative to their cancer type-specific counterparts, reflects the stabilizing effect of larger and more heterogeneous training cohorts.

Critically, when pan-cancer models were evaluated per cancer type and compared against their organ-specific equivalents, performance was preserved or improved in most settings (see **Section S1, Table S7**). For *TP53*, the pan-cancer model exceeded the cancer type-specific model in six of eight evaluated organs, with particularly notable gains in LUSC (AUC 0.98 vs. 0.61), LIHC (AUC 0.90 vs. 0.69), and HNSC (AUC 0.97 vs. 0.72), suggesting that cross-cancer supervision enriches the model with transferable morphological priors that single-organ training cannot capture. Similarly, the pan-cancer *APC* model substantially outperformed the CRC-specific model (AUC 0.91 vs. 0.71), and the *KRAS* pan-cancer model improved performance in both PAAD (AUC 0.90 vs. 0.71) and CRC (AUC 0.74 vs. 0.63). For *TTN*, pan-cancer training yielded improvements in both CRC (AUC 0.64 vs. 0.63) and STAD (AUC 0.63 vs. 0.61). The *PIK3CA* pan-cancer model improved performance markedly in BRCA (AUC 0.83 vs. 0.65), though performance in CRC declined modestly (AUC 0.62 vs. 0.64).

### Leave-one-cancer-out evaluation reveals transferable mutation-associated morphology across tumor types

**Figure 5 |:**
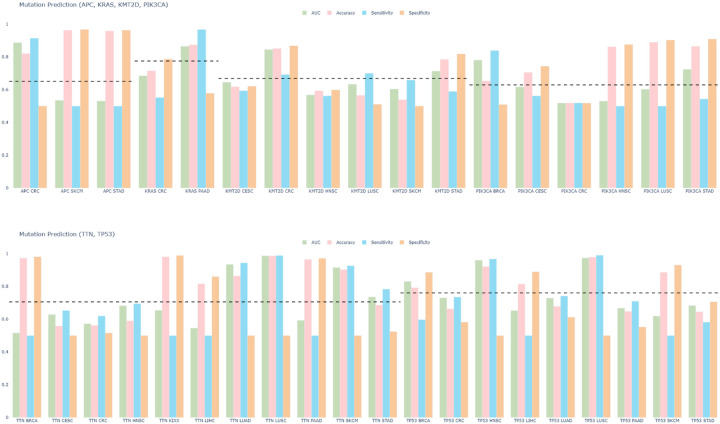
Leave-one-cancer-out evaluation demonstrates cross-tissue transferability of mutation-associated morphology. Leave-one-cancer-out (LOCO) evaluation of pan-cancer GenoGlyph models for *TP53, KRAS, APC, PIK3CA, KMT2D*, and *TTN*. For each experiment, models were trained on all cancer types except one held-out tumor type and subsequently evaluated on the excluded cohort. Bars indicated performance for each held-out cancer across different metrics. Dotted lines shows the mean AUC per mutation across different cancer types.

To further assess whether GenoGlyph captures mutation-associated morphological patterns that generalize beyond tissue-specific contexts, we performed a leave-one-cancer-out (LOCO) evaluation for the six pan-cancer gene models. In this setting, models were trained on all cancer types except one and subsequently evaluated on the held-out cancer, providing a stringent test of cross-tissue generalization and the robustness of learned genotype–phenotype relationships (Figure 5, **Table S8**).

Across genes, GenoGlyph demonstrated consistent cross-cancer transferability, supporting the presence of shared mutation-associated morphological signatures across tumor types. Among the evaluated genes, TP53 exhibited the strongest overall cross-cancer generalization, achieving a mean AUC of 0.76 ± 0.13 across 9 held-out cancer types. Notably, performance remained high in several cancers, including HNSC (AUC 0.96), LUSC (AUC 0.97), and BRCA (AUC 0.83), indicating that *TP53*-associated morphological alterations are broadly conserved across epithelial malignancies. Moderate performance was observed in CRC (AUC 0.73), LUAD (AUC 0.73), and STAD (AUC 0.68), further supporting the generalizability of *TP53*-associated histomorphological features.

*KRAS* also demonstrated strong cross-cancer transferability, with a mean AUC of 0.77 ± 0.13 across CRC and PAAD. Notably, performance remained high in pancreatic cancer (AUC 0.86), suggesting that morphological features learned from other cancers can successfully identify *KRAS*-driven phenotypes in pancreatic tumors. Similarly, *APC* exhibited moderate generalization performance (AUC 0.65 ± 0.20), with strong discrimination in CRC (AUC 0.89) but limited transferability to non-gastrointestinal cancers such as SKCM and STAD (AUC 0.54 and 0.53, respectively), where *APC* mutation prevalence is substantially lower. This pattern is consistent with Bayesian principles that govern all diagnostic tests, the model achieves higher predictive accuracy in cancers with a greater prevalence of the target mutations. Cancer types with lower mutation frequency inherently present a more challenging classification boundary, independent of the model’s capacity to learn morphological signal.

For *KMT2D*, cross-cancer evaluation yielded moderate performance (AUC 0.67 ± 0.10), with the strongest transferability observed in CRC (AUC 0.85) and STAD (AUC 0.71), but the performance was modest in squamous malignancies, including HNSC (AUC 0.57) and LUSC (AUC 0.63). *PIK3CA* also demonstrated modest cross-cancer generalization (AUC 0.63 ± 0.10), with the strongest performance observed in BRCA (AUC 0.78) and STAD (AUC 0.72), while the performance in CRC and HNSC approached near-random discrimination.

Despite being considered a passenger mutation, *TTN* demonstrated notable cross-cancer performance, achieving a mean AUC of 0.71 ± 0.17 across 11 cancer types. Particularly strong discrimination was observed in LUSC (AUC 0.99), LUAD (AUC 0.93), and SKCM (AUC 0.92), while moderate performance was observed in STAD (AUC 0.74) and HNSC (AUC 0.68).

### GenoGlyph predictions are concordant with oncogenic pathway activation

To assess whether GenoGlyph’s mutation predictions reflect genuine oncogenic pathway dysregulation rather than incidental morphological correlates, we integrated model outputs with single-sample gene set enrichment analysis (ssGSEA) scores derived from matched TCGA RNA-seq data. For each gene, biologically cognate Hallmark pathways were selected a priori. Each sample was classified into one of four prediction outcome groups, true positive (TP), false positive (FP), false negative (FN), and true negative (TN), based on agreement between GenoGlyph’s binarised output and TCGA ground-truth mutation status. Group differences in ssGSEA enrichment scores were quantified using two-sided Mann–Whitney U tests with rank-biserial correlation (r𝒯) as the effect size.

**Figure 6 |:**
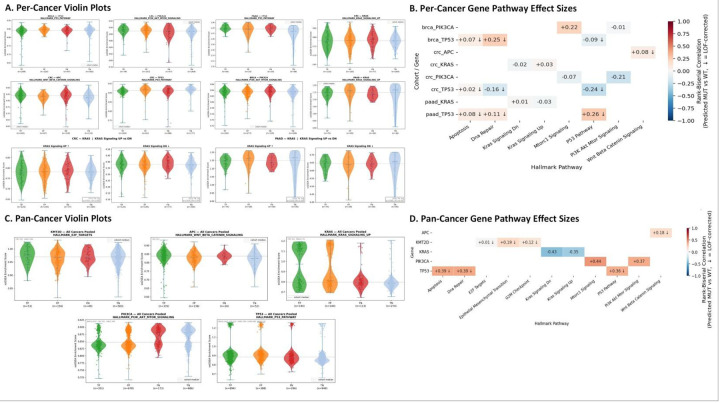
GenoGlyph mutation predictions are concordant with oncogenic pathway activity across cancer types and at pan-cancer scale. **(A)** ssGSEA Hallmark pathway enrichment scores stratified by GenoGlyph prediction outcome for eight cancer type–gene combinations. Row 1: BRCA–*TP53* (HALLMARK_P53_PATHWAY), CRC–*PIK3CA* (HALLMARK_PI3K_AKT_MTOR_SIGNALING), PAAD–*TP53* (HALLMARK_P53_PATHWAY), and CRC–*KRAS* (HALLMARK_*KRAS*_SIGNALING_UP). Row 2: CRC–*APC* (HALLMARK_WNT_BETA_CATENIN_SIGNALING), CRC–*TP53* (HALLMARK_P53_PATHWAY), BRCA–*PIK3CA* (HALLMARK_PI3K_AKT_MTOR_SIGNALING), and PAAD–*KRAS* (HALLMARK_*KRAS*_SIGNALING_UP). Row 3: Side-by-side comparison of *KRAS* Signaling UP (↑) and *KRAS* Signaling DN (↓) enrichment scores for CRC–*KRAS* (left pair) and PAAD–*KRAS* (right pair), with FP-vs-TN Mann–Whitney p-values and rank-biserial correlations annotated in each panel. Dashed lines indicate the cohort-level median enrichment score. Each dot represents one patient sample; violin width reflects the distribution density at each score level. **(B)** Directionally corrected pan-cancer heatmap of rank-biserial correlation effect sizes for predicted mutant vs. predicted wild-type comparisons (TP+FP vs. FN+TN) across eight cohort–gene pairs (rows) and nine Hallmark pathways (columns). Warm cells (red/orange) indicate concordant pathway activation or suppression relative to predicted mutation status; cold cells (blue) indicate discordant or null associations. Cells annotated with ↓ indicate loss-of-function genes (*TP53, APC*) for which the rank sign has been inverted so that concordant pathway suppression appears as a warm cell, matching the gain-of-function convention. Empty cells indicate pathway–gene combinations not tested for that cohort. **(C)** ssGSEA enrichment scores stratified by GenoGlyph prediction outcome for five pan-cancer models. Row 1: *KMT2D* (HALLMARK_E2F_TARGETS), *APC* (HALLMARK_WNT_BETA_CATENIN_SIGNALING), and *KRAS* (HALLMARK_*KRAS*_SIGNALING_UP). Row 2: *PIK3CA* (HALLMARK_PI3K_AKT_MTOR_SIGNALING) and *TP53* (HALLMARK_P53_PATHWAY). **(D)** Directionally corrected heatmap of rank-biserial correlation effect sizes for pan-cancer predicted mutant vs. predicted wild-type comparisons across five genes (rows) and eleven Hallmark pathways (columns), pooling all available cohorts per gene. Warm cells indicate concordant activation (GOF genes) or suppression (LOF genes, marked ↓).

Pan-cancer GenoGlyph predictions demonstrated robust and directionally concordant pathway associations across all five evaluated genes (Figure 6D). For *TP53*, predicted mutants showed profound and highly significant suppression of all three cognate pathways relative to predicted wild-type samples: HALLMARK_P53_PATHWAY $\left(\text{r}𝒯=-0.361,\text{p}=1.25\times 10^{-55}\right)$, HALLMARK_APOPTOSIS $\left(\text{r}𝒯=-0.389,\text{p}=2.36\times 10^{-64}\right)$, and HALLMARK_DNA_REPAIR $\left(\text{r}𝒯=-0.388,\text{p}=7.65\times 10^{-64}\right)$. The negative direction of these effects is directionally concordant with *TP53* loss-of-function biology, wherein p53 inactivation suppresses its transcriptional targets governing apoptosis and DNA damage response. For *KRAS*, predicted mutants showed significant enrichment of both HALLMARK_KRAS_SIGNALING_UP $\left(\text{r}𝒯=-0.355,\text{p}=1.97\times 10^{-16}\right)$ and HALLMARK_KRAS_SIGNALING_DN $\left(\text{r}𝒯=-0.429,\text{p}=2.58\times 10^{-23}\right)$ relative to predicted wild-type. Notably, the downstream suppression signature (DN) carried a larger effect size than the activation signature (UP), consistent with oncogenic *KRAS* simultaneously activating proliferative programs while suppressing differentiation. For *PIK3CA*, predicted mutants showed markedly elevated HALLMARK_MTORC1_SIGNALING $(\text{r}𝒯=+0.444,\text{p}=\left.3.10\times 10^{-62}\right)$ and HALLMARK_PI3K_AKT_MTOR_SIGNALING $\left(\text{r}𝒯=+0.368,\text{p}=2.91\times 10^{-43}\right)$ relative to predicted wild-type, with mTORC1 consistently providing the stronger signal across all cohorts, confirming that *PIK3CA* mutations preferentially engage the mTORC1 effector arm. For *APC*, predicted mutants showed significantly lower Wnt/β−catenin pathway scores relative to predicted wild-type $\left(\text{r}𝒯=-0.184,\text{p}=9.25\times 10^{-3}\right)$, directionally concordant with *APC* loss disinhibiting β-catenin nuclear translocation and thereby suppressing the canonical pathway readout in ssGSEA scoring, which reflects the net transcriptional output of Wnt-responsive genes (as shown in Figure 6C). For *KMT2D*, predicted mutants showed suppression of HALLMARK_G2M_CHECKPOINT $\left(\text{r}𝒯=-0.120,\text{p}=9.42\times 10^{-3}\right)$ and HALLMARK_EPITHELIAL_MESENCHYMAL_TRANSITION $\left(\text{r}𝒯=-0.188,\text{p}=4.63\times 10^{-5}\right)$, consistent with *KMT2D* loss disrupting enhancer-mediated cell cycle and epithelial identity programs. Across all five genes, concordant pathway associations were confirmed both at the cancer type–specific level and in the pooled pan-cancer analysis (Figure 6B,D), collectively demonstrating that GenoGlyph predictions are not merely correlated with genomic labels but track functionally active downstream signaling states.

### Pathway concordance provides a VUS functional annotation framework

A central challenge in clinical genomics is the interpretation of VUS, mutations catalogued by sequencing that lack evidence of functional impact. We reasoned that cases where GenoGlyph’s morphological prediction disagrees with the sequencing-derived ground truth may not represent model errors but may instead identify samples in which pathway dysregulation is present despite an ambiguous or absent genomic annotation. To test this, we examined pathway activity in FP samples and compared them against TN using the same Mann–Whitney U framework.

Across 7 FP-vs-TN comparisons reaching statistical significance (p < 0.05), 5 were directionally concordant with the expected gene biology (Figure 6A). For *TP53*, false positive samples showed significantly suppressed DNA repair pathway activity relative to true negatives in both BRCA $\left(\text{r}𝒯=-0.300,\text{p}<0.0001,{\text{n}}_{\text{FP}}=120\right)$ and PAAD $\left(\text{r}𝒯=-0.348,\text{p}=0.016,{\text{n}}_{\text{FP}}=25\right)$, as well as suppressed apoptosis signalling in BRCA (r𝒯=−0.149,p=0.010) and suppressed p53 pathway activity in PAAD (r𝒯=−0.290,p=0.045). For *PIK3CA*, false positives in BRCA showed significantly elevated mTORC1 activity relative to true negatives $\left(\text{r}𝒯=+0.172,\text{p}<0.001,{\text{n}}_{\text{FP}}=277\right)$, concordant with activating PI3K pathway biology. In each of these instances, the false positive samples’ pathway activity profile resembled that of true positive samples rather than true negatives, demonstrating that GenoGlyph’s morphological signal tracks functional pathway state independently of the binary sequencing annotation (as shown in Figure 5B).

### GenoGlyph mutation probability scores carry prognostic information across independent external cohorts

To determine whether GenoGlyph captures clinically relevant information beyond mutation classification, we evaluated the prognostic utility of mutation probability scores in four independent external cohorts comprising 982 patients with LUAD, CRC, PDAC, and metastatic HR+/HER2− BRCA (**Sections S4-S5; Figures S14-S30**). Across cohorts, several probability scores stratified patient outcomes despite being derived solely from H&E morphology. In CRC, BRAF probability demonstrated the strongest continuous prognostic associations, predicting inferior OS, DFS, and RFS after FDR correction (HR = 2.01–2.28), while KRAS probability provided the most temporally stable stratification, with significant landmark separation across all five years for all endpoints. KMT2D probability also identified CRC patients with persistently worse long-term outcomes. In LUAD, KRAS probability consistently predicted adverse OS, DFS, and RFS, whereas TP53 probability was associated primarily with recurrence-related endpoints. In PDAC, KRAS probability unexpectedly showed a strong protective association with OS, suggesting that the pan-cancer model captures prognostically favorable glandular differentiation states rather than mutation status alone in a disease where KRAS mutations are nearly ubiquitous. In metastatic BRCA, TP53 probability identified patients with markedly shorter progression-free survival characterized by late divergence, whereas PIK3CA probability showed a protective association with OS. Collectively, these findings demonstrate that GenoGlyph-derived mutation probabilities encode prognostically meaningful morphological information that generalizes across independent cohorts and clinical contexts.

## Discussion

GenoGlyph demonstrates that genotype–phenotype relationships in solid tumors are not idiosyncratic to individual cancers but constitute transferable biological signals systematically legible from H&E morphology. Across 14 cancer types and 6,391 cases, GenoGlyph substantially improves upon prior pan-cancer benchmarks [13, 38, 39] (**Table S9**), establishes the conditions under which cross-cancer training amplifies single-organ performance, and demonstrates for the first time that mutation-derived latent representations carry independent prognostic utility. A central observation is that mutation predictability is influenced by both the extent to which a mutation manifests as discernible morphological changes and the prevalence of that mutation within the population being studied.

*TP53* is the clearest illustration of the former. Strong performance across LUSC, HNSC, LIHC, and BRCA reflects *TP53* mutation’s well-established morphological consequences, including high nuclear grade, marked pleomorphism, elevated mitotic index, and loss of organized differentiation. *BRAF V600E* in CRC is similarly tractable, associated with the serrated neoplasia pathway, mucinous or poorly differentiated histology, and in the microsatellite instable subset, prominent tumor-infiltrating lymphocytes and peritumoral lymphoid reactions [40]. *CTNNB1* in LIHC reflects the well-documented composite HCC morphology driven by constitutive Wnt/β−catenin activation, with well-differentiated trabecular or pseudoglandular growth and minimal nuclear atypia defining the S3 molecular class of HCC [41].

Among the most interesting findings is that pan-cancer organ-agnostic models consistently matched or exceeded cancer type-specific counterparts, with dramatic improvements for *TP53* in LUSC (0.61 → 0.98), LIHC (0.69 → 0.90), and HNSC (0.72 → 0.97), and for *APC* in CRC (0.71 → 0.91). Three mechanisms potentially drive this effect. First, aggregating cases across cancer types dramatically expands training diversity, a *TP53* model trained on 4,107 cases spanning ten cancer types learns a far richer representation of mutation-associated morphology than one confined to 265 LUAD cases. Second, pan-cancer training acts as implicit domain adversarial regularization, i.e., the model cannot exploit organ-specific confounders as mutation proxies because the same mutation status must be discriminated across architecturally heterogeneous contexts, forcing genuinely mutation-associated rather than tissue-associated features. Third, cross-cancer morphological priors transfer effectively, such as high-grade nuclear morphology and increased mitotic activity learned as *TP53* correlates in BRCA function as informative priors when evaluating LUSC, where the signal exists but has lower single-cancer signal-to-noise. The LOCO evaluation provides the strongest evidence for this cross-tissue generalizability. A model trained on all cancer types except LUSC predicts *TP53* mutation in LUSC with AUC 0.97, since *TP53*’s downstream effects are universal cellular alterations to p53 dysfunction transcending tissue identity. *KRAS* similarly demonstrated strong LOCO generalization (mean AUC 0.77 ± 0.13), confirming that *KRAS*-associated desmoplastic and cytological features are not simply reflections of organ-specific biology. By contrast, *APC* showed strong LOCO performance within gastrointestinal contexts (CRC: 0.89) but markedly lower transferability to non-gastrointestinal cancers (~0.53), consistent with *APC*’s tissue-specific developmental role in intestinal homeostasis. Collectively, the LOCO results support a hierarchical model of genotype–phenotype universality that some mutations produce pan-tumoral cellular vocabulary (*TP53, KRAS*), while others encode tissue-specific morphological dialects (*APC*) whose signal weakens outside their native context.

The prediction of *TTN* mutations warrants separate consideration. As the longest gene in the human genome, *TTN* accumulates passenger mutations proportionally to overall somatic mutational burden, with *TTN* mutation count representing among the strongest single-gene surrogates for tumor mutation burden (TMB) by whole-exome sequencing (p = 0.917 pan-cancer) [53]. GenoGlyph’s pan-cancer AUC of 0.73 ± 0.01 for *TTN* can therefore be best interpreted as morphological TMB prediction. learning tumor-infiltrating lymphocyte density, immune infiltration patterns, and architectural disorganization characteristic of hypermutated tumors, consistent with dedicated H&E TMB prediction models achieving comparable AUCs [54, 55].

The pathway analyses provide important biological validation of GenoGlyph’s mutation predictions. Across multiple driver genes and cancer types, morphologically inferred mutation states consistently corresponded to expected downstream transcriptional programs, demonstrating that the model captures functionally active oncogenic states rather than merely correlating with sequencing labels. Importantly, this biological concordance extended beyond correctly classified samples. Several sequencing-discordant predictions exhibited pathway activity patterns resembling true positives rather than true negatives. For example, BRCA *TP53* false positives showed suppressed DNA repair (rb = −0.300, p < 0.0001) and apoptosis signaling (rb = −0.149, p = 0.010), while PAAD *TP53* false positives exhibited suppressed DNA repair (rb = −0.348, p = 0.016) and p53 pathway activity (rb = −0.290, p = 0.045), all directionally concordant with *TP53* loss-of-function biology. These findings suggest that histomorphology may encode functional consequences of genomic alterations even when they are incompletely represented by binary mutation annotations. Rather than constituting classifier errors, some sequencing-discordant predictions may represent clinically relevant states in which pathway dysregulation arises through mechanisms not captured by conventional variant-calling pipelines, including protein-level inactivation, deep intronic or regulatory variants, subclonal mutations below detection thresholds, or convergent genomic events such as PTEN loss activating PI3K signaling. In this context, GenoGlyph may function as a morphology-based functional genomics tool, using H&E-derived phenotypes to identify cases warranting deeper molecular interrogation rather than reflexive reclassification as model failures. Two exceptions, elevated PI3K/AKT/mTOR activity in *PIK3CA* false positives in CRC (r𝒯=−0.191,p=0.001) and elevated DNA repair activity in *TP53* false positives in CRC (r𝒯=+0.234,p=0.003), were directionally discordant with expected biology and may reflect background PI3K activation through APC/Wnt crosstalk and compensatory DNA repair upregulation in microsatellite-stable tumors, respectively. These anomalous findings highlight the need for prospective functional validation and should be regarded as limitations rather than evidence of model confabulation. Collectively, these observations indicate that GenoGlyph learns latent representations of tumor biology that extend beyond genotype itself and are tightly coupled to downstream cellular programs, supporting the use of morphology as a surrogate readout of oncogenic signaling.

The clinical utility of GenoGlyph operates across two complementary dimensions. The most immediate is providing probabilistic genomic characterization from the same H&E slide used for routine diagnosis, in settings where NGS is unavailable, delayed, or tissue-limited. GenoGlyph probability scores can triage patients into high- and low-probability mutation strata, concentrating sequencing capacity on cases where confirmation has greatest therapeutic consequence [43], a strategy shown to reduce sequencing burden by up to 49.9% while maintaining 95% sensitivity for clinically actionable mutations [44]. The second and arguably more novel dimension is prognostic stratification directly from H&E morphology, without any molecular testing. Although the present analyses focus on morphology-derived representations alone, GenoGlyph could potentially be integrated with additional clinical variables, molecular biomarkers, and treatment information to enable more comprehensive risk models and further improve patient outcome prediction. Our survival analyses confirm that GenoGlyph’s genomic-aware representations carry clinically meaningful prognostic signal across four independent external cohorts. In CRC, *BRAF* probability stratifies patients with FDR-robust OS, DFS, and RFS differences (HR 2.01–2.28), consistent with meta-analytic literature reporting pooled *BRAF* mutation HR of 2.25 for OS across 26 studies [40, 45, 46]. *KRAS* probability in CRC achieves landmark-persistent stratification at every timepoint from 1 through 5 years (5yr OS: 69.7% vs. 82.0%, p < 0.01), with HR of 1.55–1.68 directionally consistent with meta-analytic estimates of HR 1.63 for DFS in microsatellite-stable CRC [45], attenuated, as expected, by imperfect mutation classification from morphology. Notably, the discordance between *BRAF*’s significant continuous Cox associations and non-significant binary analyses illustrates that dichotomization of probability scores discards graded signal precisely where continuous measurement is most informative. In PAAD, the strongly protective association between *KRAS* probability and OS (HR = 0.09, p < 0.0001; landmark significance at every timepoint, 4yr p < 0.0001) demands biological contextualization rather than dismissal. As an early driver event in PAAD carcinogenesis, *KRAS* is mutated in ~90% of PAADs, limiting its utility as a binary biomarker. The pan-cancer GenoGlyph model instead learns the broader morphological continuum of *KRAS*-associated glandular differentiation and, in PAAD, may capture aspects of the classical–basal differentiation axis: the classical subtype, defined by well-differentiated glandular morphology and *GATA6* expression, carries substantially better prognosis (median OS ~16 vs. ~11 months) and greater FOLFIRINOX benefit than the poorly differentiated basal-like subtype [47, 48]. This interpretation is consistent with PanSubNet’s independent demonstration that classical and basal-like subtypes manifest as morphologically learnable H&E patterns [49–52]. Direct validation against Moffitt subtype labels available for a subset of this cohort revealed that *KRAS* probability scores did not discriminate classical from basal-like cases above chance level. This dissociation suggests the model is not functioning as a straightforward subtype classifier; rather, it may be capturing a finer-grained morphological gradient correlated with prognosis that partially overlaps with, but is not reducible to, the binary Moffitt distinction. We therefore propose a more nuanced principle that pan-cancer mutation models applied to cancers with near-universal mutation prevalence can learn morphological gradients with independent prognostic relevance, without necessarily recapitulating established discrete subtype boundaries. In metastatic HR+/HER2− BRCA, *TP53* probability predicts PFS with a striking late-divergence pattern, no difference at year 1, catastrophic separation by year 4 (0% vs. 25% progression-free), consistent with *TP53*-mutated HR+ tumors exhibiting delayed endocrine resistance under CDK4/6 inhibitor regimens. *PIK3CA* probability shows a nominally significant protective OS association in BRCA (HR 0.61, p = 0.044), biologically plausible given *PIK3CA*-mutated HR+ tumors’ differential sensitivity to alpelisib-based therapy.

Beyond its utility as a discovery tool, the scalable architecture of GenoGlyph offers immediate translational utility for clinical workflows, particularly as an automated triage mechanism within digitized pathology ecosystems. In standard oncology pipelines, NGS routinely incurs a multi-week turnaround bottleneck, a delay that can be critical for patients with aggressive, rapidly progressing malignancies. Running continuously in the background of digital scanning platforms, GenoGlyph can instantly analyze standard diagnostic slides at the point of care, flagging high-probability actionable alterations (e.g., *TP53, KRAS*, or *PIK3CA*) and providing immediate clinical risk stratification. This rapid, automated prescreening allows pathology departments to fast-track high-risk cases for immediate multidisciplinary tumor board review and preemptively optimize clinical trial matching, drastically shrinking the time from initial biopsy to targeted therapeutic intervention. Furthermore, in resource-limited or community healthcare settings where NGS infrastructure is financially or logistically prohibitive, GenoGlyph can act as an accessible, cost-effective surrogate, democratizing precision oncology by delivering immediate molecular insights anywhere an optical scanner is available.

Crucially, GenoGlyph’s clinical utility extends to capturing critical biological context that standard bulk sequencing inherently overlooks. In clinical practice, approximately 10% to 20% of tissue biopsies fail molecular sequencing due to low tumor purity, DNA degradation, or insufficient tissue volume, often categorized as Quantity Not Sufficient (QNS). Because GenoGlyph relies exclusively on standard diagnostic slides, it provides a vital diagnostic safety net, extracting latent feature representations and rescuing genomic insights for cohorts that would otherwise remain molecularly uncharacterized. Moreover, because bulk NGS homogenizes tissue samples, it completely strips away the spatial architecture of the tumor microenvironment. By mapping GenoGlyph’s cell-level mutational predictions directly back onto the WSI, clinicians can visually resolve intratumoral heterogeneity. This enables the spatial mapping of distinct subclones and their localized interactions with the immune stroma, providing a granular, biologically grounded canvas to better understand therapeutic resistance and tumor evolution.

Several limitations may constrain interpretation. Training and cross-validation were performed exclusively on TCGA data comprising predominantly resected, formalin-fixed, paraffin-embedded specimens, and generalization to biopsy-derived or cytological material requires prospective external validation. Survival analyses are retrospective and univariable, precluding adjustment for tumor stage, treatment regimen, or performance status; multivariable validation in prospective cohorts is required before clinical application. The BRCA survival cohort (n = 94) is underpowered, and findings there should be regarded as hypothesis-generating. The pathway association and VUS analyses, while mechanistically informative, are based on cohort-level transcriptomic averages and cannot resolve intra-tumor heterogeneity or confirm functionality at the individual variant level; prospective functional annotation studies are required before applying this framework to clinical variant interpretation.

Together, these findings establish that the histopathological language of cancer encodes genomic state with sufficient fidelity to support pan-cancer mutation inference, biological characterization, and prognostic stratification directly from routine H&E sections. Rather than functioning solely as a surrogate for sequencing, GenoGlyph learns representations coupled to downstream oncogenic programs and clinically meaningful tumor states, enabling morphology to serve as a scalable and biologically grounded substrate for precision oncology. As computational pathology infrastructure matures, such approaches may help extend molecularly informed cancer care beyond the practical constraints of genomic testing.

## Methods

**Figure 7 |:**
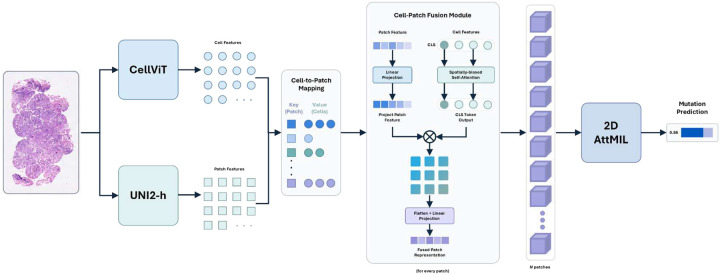
Architecture of the GenoGlyph framework for multi-scale histopathological representation learning. Detailed architecture of the GenoGlyph framework. Whole-slide images were processed using a dual-scale pipeline designed to capture both tissue architecture and fine-grained cellular morphology. At 20× magnification, WSIs were tiled into non-overlapping image patches and encoded using the UNI2-h pathology foundation model to generate patch-level architectural embeddings. At 40× magnification, individual nuclei were segmented and classified using CellViT++, producing cell-level embeddings and spatial centroid coordinates for neoplastic, stromal, inflammatory, benign epithelial, and dead cells. Cells were mapped to their corresponding tissue patches based on centroid location. Within each patch, cellular embeddings were aggregated through a spatially biased self-attention mechanism incorporating a learnable CLS token and distance-aware attention penalties to model biologically coherent cellular neighborhoods. Cross-scale fusion between cellular and tissue-level representations was performed using an outer-product interaction module, enabling multiplicative integration between cellular morphology and architectural context. Fused patch representations were subsequently aggregated at the slide level using a multi-dimensional attention-based multiple instance learning (AttMIL) framework employing higher-dimensional attention projections to improve representation expressivity. The final slide-level embedding was passed through a classification head to generate mutation probability predictions. The framework was trained end-to-end using binary cross-entropy optimization with harmonized sensitivity-specificity early stopping criteria.

GenoGlyph is built upon the PathRosetta [36] framework originally developed for recurrence prediction in invasive lung adenocarcinoma. Inspired by PathRosetta’s explicit cellular- and tissue-level modeling paradigm, GenoGlyph incorporates several architectural refinements to improve efficiency, scalability, and robustness. In addition, GenoGlyph is evaluated on a substantially more complex predictive task involving larger and more heterogeneous datasets, increased biological and technical variability, and broader validation settings to assess generalizability across these challenges. Detailed architectural components of GenoGlyph are described below (see Figure 7).

### Multi-Scale Feature Extraction

WSI preprocessing followed a dual-scale strategy designed to capture cellular morphology and tissue architecture simultaneously. At 20× magnification, each WSI was tiled into non-overlapping patches of 256 × 256 pixels. Patch-level feature vectors were extracted using UNI2-h, a pathology foundation model [56]. Formally, for patch p, the patch embedding is:

$$e_{p}=f_{\text{patch}}\left(p;\theta_{UNI}\right)\in R^{1536}$$

where $f_{\text{patch}}\left(p;\theta_{UNI}\right)$ denotes the UNI2-h encoder with frozen weights $\theta_{UNI}$. At 40× magnification, cell nuclei were segmented and classified into five categories, neoplastic, stromal, inflammatory, benign epithelial, and dead cells, using CellViT++, a ViT-based cell segmentation and classification framework [57, 58]. For each cell i, CellViT++ yields a cell embedding and centroid coordinates:

$$c_{i}=f_{\text{cell}}\left(x_{i};\theta_{\text{CellViT}}\right)\in R^{1280},\;\left(x_{i},y_{i}\right)=\text{centroid}(i)$$

where $x_{i}$ is the image region centered on cell nucleus i. This dual-scale design ensures that fine-grained nuclear morphology and broad architectural context are encoded as complementary representations.

### Cell-to-Patch Mapping and Spatially Biased Cell Attention

To link cellular and tissue-level representations, each cell was mapped to the patch containing its centroid. For patch p, the corresponding cell set is:

$$C_{p}=\left\{c_{i}\in C|\left(x_{i},y_{i}\right)\in p\right\}$$

where C is the full set of detected cells. Patch embeddings were linearly projected from $R^{1536}$ to $R^{768}$ via a learned projection matrix $W_{p}\in R^{\left\{768\times 1536\right\}}$:

$${\tilde{\text{e}}}_{p}=W_{p}\cdot e_{p}\in R^{768}$$

Within each patch, the set of cell embeddings $\left\{c_{1},\ldots ,c_{\left\{\left|c_{p}\right|\right\}}\right\}$ was aggregated using a self-attention mechanism augmented with a learnable CLS token $e_{\text{CLS}}\in R^{\left\{1280\right\}}$. The input sequence is $X_{p}=\left[e_{\text{CLS}},c_{1},\ldots ,c_{\left\{\left|c_{p}\right|\right\}}\right]\in R^{\left\{(n+1)\times 1280\right\}}$. Queries, keys, and values are computed as:

$$Q=W_{Q}\cdot X_{p},\;K=W_{K}\cdot X_{p},\;V=W_{V}\cdot X_{p}$$

where $W_{Q},W_{K},W_{V}\in R^{\left\{768\times 1280\right\}}$. Raw attention scores are computed as:

$$A_{raw}(i,j)=\frac{\left(Q_{i}\cdot K_{j}^{T}\right)}{\sqrt{d_{K}}}$$

where $d_{K}=768$ is the key dimension. A spatial bias is then applied by penalizing attention between spatially distant cells:

$$A_{\text{spatial}}(i,j)=A_{\text{raw}}(i,j)-\text{dist}\left(\left(x_{i},y_{i}\right),\left(x_{j},y_{j}\right)\right)$$

where dist(⋅,⋅) is the Euclidean distance between cell centroids in pixel space. This spatial bias encodes the biological principle that spatially proximate cells form coherent functional neighborhoods. The final attention weights and output are:

$$A=\text{softmax}\left(A_{\text{spatial}}\right),\;Z=A\cdot V$$

The updated CLS token $z_{\text{CLS}}\in R^{\left\{768\right\}}$, extracted from the first row of Z, carries an aggregated cellular representation weighted by both morphological content and spatial proximity.

### Cross-Scale Fusion via Outer Product

Cellular and patch-level representations were fused via an outer product operation, capturing all pairwise multiplicative interactions between the two embedding spaces. For patch p, the fused representation is:

$$F_{p}={\tilde{\text{e}}}_{p}⊗z_{CLS}\in R^{\left\{768\times 768\right\}}$$

This outer product matrix is flattened and projected back to $R^{768}$ through a learned projection:

$$f_{p}=W_{\text{fusion}}\cdot \text{flatten}\left(F_{p}\right),\;W_{\text{fusion}}\in R^{\left\{768\times 768^{2}\right\}}$$

where $f_{p}\in R^{768}$ is the final fused patch representation encoding both intrinsic cellular morphology and the architectural tissue context. This interaction representation is substantially richer than additive or concatenation-based fusion, as it encodes the full multiplicative cross-talk between cellular and architectural feature dimensions.

### Multi-Dimensional Attention-Based Multiple Instance Learning

The fused patch representations $\left\{f_{1},\ldots ,f_{K}\right\}\in R^{768}$ from a WSI constitute a bag of K instances. Slide-level aggregation was performed using attention-based multiple instance learning (AttMIL). To overcome the expressivity limitations of standard 1D attention projection, we introduced multi-dimensional attention projections onto 2D and 3D subspaces. The generalized attention weight for instance k is:

$$\alpha_{k}=\frac{\text{exp}\left\{{\left\|W^{\text{T}}\left(\text{tanh}\left(Vf_{k}^{T}\right)⊙\text{sigmoid}\left(Uf_{k}^{T}\right)\right)\right\|}_{F}\right\}}{\sum_{j=1}^{K}\text{exp}\left\{{\left\|W^{\text{T}}\left(\text{tanh}\left(Vf_{j}^{T}\right)⊙\text{sigmoid}\left(Uf_{j}^{T}\right)\right)\right\|}_{F}\right\}}$$

where $V,U\in R^{\left\{L\times 768\right\}}$ are learned projection matrices, $W\in R^{\left\{L\times d\right\}}$ with d∈{1,2,3} for 1D, 2D, and 3D projections respectively, σ(⋅) denotes the sigmoid function, ⊙ denotes element-wise multiplication, and $\|\cdot {\|}_{F}$ is the Frobenius norm. For scalar d=1, this reduces to standard AttMIL; for d>1, the attention score is computed over a richer multi-dimensional projection space, capturing interaction patterns among feature dimensions that a single weight vector cannot discriminate. The slide-level representation is the attention-weighted sum:

$$h_{\text{slide}}=\Sigma_{k}\alpha_{k}\cdot f_{k}\in R^{768}$$

and the final mutation probability is produced by a linear classifier followed by sigmoid activation:

$$\hat{\text{y}}=\sigma \left(w^{T}h_{\text{slide}}+b\right)$$

### Training and Optimization

All models were trained using five-fold patient-stratified cross-validation on NVIDIA A100 GPUs for up to 100 epochs. Optimization used the Adam optimizer with learning rate $\eta =5\times 10^{-5}$ and weight decay $\lambda =1\times 10^{-5}$. The training objective was binary cross-entropy with L2 regularization:

$$L=-[y\;log(\hat{\text{y}})+\left(1-y\right)log(1-\hat{\text{y}})]+\lambda \|\theta {\|}^{2}$$

To ensure clinically balanced predictions, early stopping was governed by a harmonized criterion maximizing the harmonic mean of sensitivity (Sen) and specificity (Spe) on the held-out validation fold, subject to a minimum AUC threshold δ:

$$H=2\cdot \text{Sen}\cdot \text{Spe}/(\text{Sen}+\text{Spe}),\;\text{subject}\;\text{to}\;H_{\text{val}}\geq \delta$$

The model checkpoint corresponding to the epoch with the highest H satisfying the constraint was selected for evaluation. Pan-cancer models were trained identically, with cancer type identity used for stratified sampling to ensure balanced representation across tumor types within each mini-batch.

### Transcriptomic pathway association analysis

To assess whether GenoGlyph’s mutation predictions reflect genuine oncogenic pathway dysregulation, we integrated model outputs with single-sample gene set enrichment analysis (ssGSEA) scores computed from matched RNA-seq data. For each cohort, ssGSEA enrichment scores were computed using the GSVA package against the MSigDB Hallmark gene set collection v2023.1, generating a per-sample enrichment score in [0, 1] for each of 50 curated biological pathways. For each gene model, we selected the biologically cognate Hallmark pathways a priori: HALLMARK_P53_PATHWAY, HALLMARK_APOPTOSIS, and HALLMARK_DNA_REPAIR for TP53; HALLMARK_*KRAS*_SIGNALING_UP and HALLMARK_*KRAS*_SIGNALING_DN for *KRAS*; HALLMARK_WNT_BETA_CATENIN_SIGNALING for *APC*; HALLMARK_PI3K_AKT_MTOR_SIGNALING and HALLMARK_MTORC1_SIGNALING for *PIK3CA*; and HALLMARK_E2F_TARGETS, HALLMARK_G2M_CHECKPOINT, and HALLMARK_EPITHELIAL_MESENCHYMAL_TRANSITION for *KMT2D*. No pathway was assigned for *TTN*, as it is a passenger mutation without a cognate signaling program. For the pan-cancer analysis, predictions from all cancer types with available RNA-seq data were pooled per gene.

Each sample was classified into one of four prediction outcome categories based on agreement between the binarised GenoGlyph output and ground-truth mutation status from TCGA: true positive (TP; predicted mutant, truly mutated), false positive (FP; predicted mutant, truly wild-type), false negative (FN; predicted wild-type, truly mutated), and true negative (TN; predicted wild-type, truly wild-type). For samples appearing in multiple cross-validation folds, the prediction from the highest fold index was retained to ensure each sample contributed once. Patients were matched between prediction files and ssGSEA scores using the first 12 characters of the TCGA barcode; only patients present in both datasets were included.

Group differences in ssGSEA enrichment scores between outcome categories were assessed using two-sided Mann-Whitney U tests. We performed six pre-specified comparisons per pathway per cohort: TP vs FP, TP vs TN, FP vs TN, FN vs TN, predicted MUT (TP+FP) vs predicted WT (FN+TN), and true MUT (TP+FN) vs true WT (FP+TN). Effect sizes were quantified as rank-biserial correlations (rb), computed as rb = 1 - (2U)/(n_1_n_2_), ranging from −1 to +1; for loss-of-function genes (*TP53, APC, KMT2D*), the predicted-mutant group is expected to show lower pathway activity, so a negative rb indicates a concordant result. For the pan-gene directional-correction heatmap, rb values for LOF genes were sign-inverted so that concordant pathway suppression appears as a positive (warm) cell, matching the gain-of-function convention; corrected cells are annotated with ↓. No multiple testing correction was applied within the pathway association analysis, as the comparisons were pre-specified and hypothesis-driven; all reported p-values are two-sided and uncorrected.

For the variant of uncertain significance (VUS) annotation analysis, we filtered to FP-vs-TN comparisons with p < 0.05 and assigned a functional interpretation based on the direction of the effect relative to the expected gene biology: for loss-of-function genes, concordance requires FP samples to show lower pathway activity than TN (i.e., rb < 0); for gain-of-function / activating genes, concordance requires FP samples to show higher pathway activity than TN (rb > 0). Discordant findings were flagged as potentially arising from co-occurring genomic events or pathway confounders rather than direct gene function. Statistical analyses were performed in Python 3.10 using scipy 1.11 and pandas 2.0. Violin plots were generated with matplotlib 3.8, with individual data points overlaid as jittered scatter plots.

### Prognostic Survival Analysis

To assess whether learned mutation probability scores carry independent prognostic information, univariable survival analyses were performed on four external cohorts. For each gene g and cohort, the predicted probability $p_{g}\in [0,1]$ was used as a continuous prognostic variable in a Cox proportional hazards model:

$$h\left(t|p_{g}\right)=h_{0}(t)\cdot exp\left(\beta_{g}\cdot p_{g}\right)$$

where $h_{0}(t)$ is the baseline hazard and $\beta_{g}$ is the estimated log-hazard coefficient. The concordance index (C-index) was computed using the negated probability $\left(-p_{g}\right)$ as the risk score, such that C-index > 0.5 indicates higher mutation probability associating with worse survival. Binary stratification used a median probability cut, and overall log-rank tests and landmark log-rank tests at 1, 2, 3, 4, and 5 years were computed for each gene–cohort–endpoint combination. Multiple testing correction was applied using the Benjamini–Hochberg false discovery rate procedure per outcome group. Statistical analyses were performed using Python 3.10 with *lifelines* 0.28 and *statsmodels* 0.14. A p-value < 0.05 was considered statistically significant.

## Supplementary Material

Supplementary Files

This is a list of supplementary files associated with this preprint. Click to download.

- SupplementaryMaterialsGenoGlyph.docx

## Data Availability

The processed data and underlying code for this study will be made available upon reasonable request to the corresponding author. WSIs for TCGA are publicly available through Genomics Data Common portal (https://portal.gdc.cancer.gov/). Gene mutation prediction labels for TCGA patients are publicly available by Kather et al at (https://github.com/jnkather/DeepHistology). Project website is hosted at https://ai4path-lab.github.io/GenoGlyph/.
